# Multidisciplinary ecosystem to study lifecourse determinants and prevention of early-onset burdensome multimorbidity (MELD-B) – protocol for a research collaboration

**DOI:** 10.1177/26335565231204544

**Published:** 2023-09-25

**Authors:** Simon DS Fraser, Sebastian Stannard, Emilia Holland, Michael Boniface, Rebecca B Hoyle, Rebecca Wilkinson, Ashley Akbari, Mark Ashworth, Ann Berrington, Roberta Chiovoloni, Jessica Enright, Nick A Francis, Gareth Giles, Martin Gulliford, Sara Macdonald, Frances S Mair, Rhiannon K Owen, Shantini Paranjothy, Heather Parsons, Ruben J Sanchez-Garcia, Mozhdeh Shiranirad, Zlatko Zlatev, Nisreen Alwan

**Affiliations:** 1 School of Primary Care, Population Sciences and Medical Education, Faculty of Medicine, 12211 Southampton General Hospital, Southampton, UK; 2 School of Electronics and Computer Science, 7423 University of Southampton, Southampton, UK; 3 School of Mathematical Sciences, 7423 University of Southampton, Southampton, UK; 4 15577 Southampton City Council, Southampton, UK; 5 Population Data Science, Swansea University Medical School, Faculty of Medicine, Health and Life Science, 7759 Swansea University, Swansea, UK; 6 School of Life Course and Population Sciences, King’s College London, London, UK; 7 Department of Social Statistics and Demography, 7423 University of Southampton, Southampton, UK; 8 Computing Science, 3526 University of Glasgow, Glasgow, UK; 9 Public Policy Southampton, 7423 University of Southampton, Southampton, UK; 10 School of Health and Wellbeing, General Practice and Primary Care, 3526 University of Glasgow, Glasgow, UK; 11 School of Medicine, Medical Sciences and Nutrition, 1019 University of Aberdeen, Aberdeen, UK; 12 NHS Grampian Health Board, Aberdeen, UK; 13 Patient and Public Involvement and Engagement, 7425 University Hospital Southampton NHS Foundation Trust, Southampton, UK; 14 7423 The Alan Turing Institute, London, UK; 15 12211 NIHR Southampton Biomedical Research Centre, Southampton, UK; 16 NIHR Applied Research Collaboration Wessex, Southampton, UK

**Keywords:** Life course, multimorbidity, long-term conditions, health, burdensome, complex, artificial intelligence, birth cohorts, routine healthcare datasets, prevention

## Abstract

**Background:**

Most people living with multiple long-term condition multimorbidity (MLTC-M) are under 65 (defined as ‘early onset’). Earlier and greater accrual of long-term conditions (LTCs) may be influenced by the timing and nature of exposure to key risk factors, wider determinants or other LTCs at different life stages. We have established a research collaboration titled ‘MELD-B’ to understand how wider determinants, sentinel conditions (the first LTC in the lifecourse) and LTC accrual sequence affect risk of early-onset, burdensome MLTC-M, and to inform prevention interventions.

**Aim:**

Our aim is to identify critical periods in the lifecourse for prevention of early-onset, burdensome MLTC-M, identified through the analysis of birth cohorts and electronic health records, including artificial intelligence (AI)-enhanced analyses.

**Design:**

We will develop deeper understanding of ‘burdensomeness’ and ‘complexity’ through a qualitative evidence synthesis and a consensus study. Using safe data environments for analyses across large, representative routine healthcare datasets and birth cohorts, we will apply AI methods to identify early-onset, burdensome MLTC-M clusters and sentinel conditions, develop semi-supervised learning to match individuals across datasets, identify determinants of burdensome clusters, and model trajectories of LTC and burden accrual. We will characterise early-life (under 18 years) risk factors for early-onset, burdensome MLTC-M and sentinel conditions. Finally, using AI and causal inference modelling, we will model potential ‘preventable moments’, defined as time periods in the life course where there is an opportunity for intervention on risk factors and early determinants to prevent the development of MLTC-M. Patient and public involvement is integrated throughout.

## Background

A growing number of people are living with multiple long-term condition multimorbidity (MLTC-M). Factors such as demographic characteristics (e.g. age, ethnicity), lifecourse events (e.g. infections, accidents), behaviours (e.g. smoking, diet) and broader experiences (e.g. the environment people grew up in, their education, work, income) influence the chances of developing MLTC-M. MLTC-M occurs earlier in the lifecourse among people from more socioeconomically and demographically disadvantaged backgrounds.^1^ The burden of MLTC-M, and the sequence that people develop conditions, also vary. To meet the significant challenge of preventing early-onset, burdensome and complex MLTC-M there is a need to clarify the meaning of burdensomeness and complexity and, taking a lifecourse approach, to understand the influence of wider determinants (such as social, economic, and environmental factors), the role of sentinel conditions and the sequence of long-term condition (LTC) accrual. We have therefore designed a research collaboration titled ‘MELD-B’ to harness the potential of artificial intelligence (AI) to handle the complexity of analyses required to discover insights from complex national social and routine datasets across the lifecourse.

### Long term condition accrual and sentinel conditions

Earlier and greater accrual of long-term conditions (LTCs) may be influenced by the timing and nature of the exposure to key risk factors, wider determinants or other LTCs at different life stages. However, existing studies have limited time frames (5-20 years of follow up mostly during adulthood) and limited analyses of LTC accrual sequence. The sequence of a) risk factors and b) LTCs may be a key determinant of the nature and burden of LTC clusters.^2–5^

In earlier developmental work we introduced the term ‘sentinel condition’ to describe the first LTC that an individual develops in their lifecourse as part of a subsequent MLTC-M cluster.^6^ The timing and nature of sentinel conditions may influence subsequent clusters, which then influence the nature and risk of burden and outcomes. Clinical diagnosis leads to actions such as medication, specialist referral, and self-management advice. Such actions affect future trajectories. The time point of diagnosis and associated actions therefore also become important determinants of future MLTC-M, and this will be explored within the MELD-B collaboration.^2,3^

### Lifecourse determinants of MLTC-M

Developmental Origins of Health and Disease (DOHaD) has become an established research field linking aetiology of disease in adulthood with environmental exposures in utero and early life.^7^ Preconception and pregnancy are important periods and the concept of ‘fetal programming’ has emerged whereby a stimulus or insult during that period can have permanent effects on structure, physiology and metabolic system of offspring.^8–12^ Epigenetics is a biological pathway underlying DOHaD, where permanent effects of transient environmental influences alter epigenetic gene regulation.^12,13^ Socioeconomic disadvantage is key in shaping developmental life experiences.^14^ Analyses in the Hertfordshire cohort study showed that paternal social class was associated with future multimorbidity.^15^ In the Aberdeen Children of the 1950s (ACONF) cohort, lower father’s social class at birth was associated with early-onset multimorbidity.^16^ In the 1970 British Cohort Study (BCS70) those with fathers from unskilled occupational groups (vs. professional) at birth had 43% higher risk of early-onset multimorbidity.^17^ Recent policy has also focused on the importance of early life in shaping health and disease. The 2019 Health and Social Care Select Committee report described how “The first 1000 days of life, from conception to age 2, is a critical phase during which the foundations of a child’s development are laid.”^18^ Recommendations from research commissioned by the Royal Foundation of the Duke and Duchess of Cambridge included the importance of promoting education and supported wide dissemination of evidence on the primacy of the early years.^19^ Despite previous research evidence, there remains a need to explore wider early-life determinants, defined as the period from pre-conception until age 18, on the combinations of LTCs.

### Burden and complexity

MLTC-M analyses would be greatly enhanced by a better understanding of burdensomeness and complexity, and what they mean to patients and carers. MLTC-M is commonly defined as having two or more LTCs, but there is a need to move away from LTC counts towards a more sophisticated understanding of MLTC-M, considering the interplay between wider social determinants and disease, the influence of mental and physical conditions, and the importance of disease stage/severity. Conceptualisation of some of the challenges experienced by patients is encapsulated in the ‘cumulative complexity model, which includes the concept of ‘treatment burden, patient workload and ‘capacity’, and was included in the National Institute for Health and Care Excellence multimorbidity guidance.^5,20–23^ For people with greater clinical complexity and fewer available resources, total workload might outweigh ‘capacity’ (ability to manage workload conferred by treatment and the demands of everyday life) and risk treatment failure.^24^ High treatment burden is associated with poor quality-of-life and lower treatment adherence, potentially leading to worse outcomes and health service inefficiency.^25–29^ In a cross sectional study of 835 people with multimorbidity, higher treatment burden was associated with younger age (people aged 55-64 vs. those over 65).^30^ In MELD-B we will consider a broad scope of ‘burdensomeness’ and ‘complexity’ that includes treatment burden as well as disease burden and broader psychosocial factors.

## Aim

Our aim is to identify critical periods in the lifecourse for prevention of early-onset (under 65), burdensome MLTC-M, identified through the analysis of birth cohorts and electronic health records. We will use artificial intelligence (AI)-enhanced epidemiological analysis and disseminate our findings to policy makers,establishing pathways to policy and practice impact.

## Research plan/methods

### Study design

**Figure 1.**
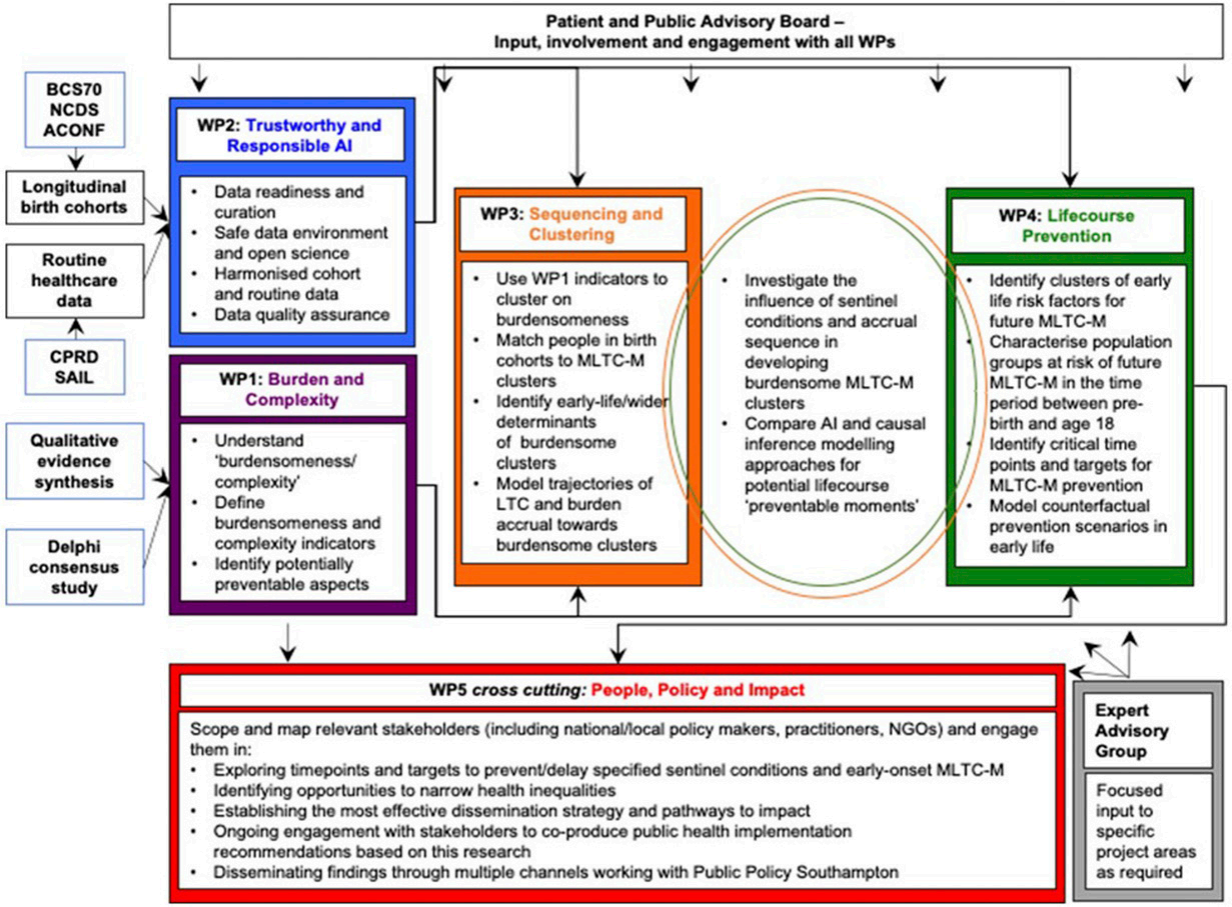
Study work package structure.

The study uses a mixed methods approach that combines qualitative evidence synthesis and quantitative analysis of birth cohorts and electronic health records. This will be achieved through five complementary work packages, a patient and public advisory board, and an expert advisory group, as shown in Figure 1.

### Research objectives

Work Package 1 - ‘Burden and complexity’:

1.1 Develop a deeper understanding of what ‘burdensomeness’ and ‘complexity’ mean to people living with early-onset MLTC-M, their carers and healthcare professionals.
1.2 Produce a suite of burdensomeness/complexity indicators for routine healthcare data as burdensomeness/complexity domains for use in clustering and clinical practice.

Work Package 2 - ‘Trustworthy and Responsible AI’:

2.1 Provide the safe data environment and readiness for AI analyses across routine healthcare data (Secure Anonymised Information Linkage (SAIL) Databank and Clinical Practice Research Datalink (CPRD)) and birth cohort data (National Child Development Study (NCDS), Aberdeen Children of the 1950s (ACONF), 1970 British Cohort Study (BCS70)).
2.2 Harmonise specified LTCs across birth cohorts and routine data.

Work Package 3 ‘Sequencing and Clustering’:

3.1 Use the burdensomeness/complexity indicators and apply AI methods to identify novel burdensome early-onset MLTC-M clusters in routine data.
3.2 Develop and apply semi-supervised learning to match individuals in birth cohorts into the routine data MLTC-M clusters and identify early-life and later determinants of the burdensome clusters using the matched datasets.
3.3 Describe and model trajectories of LTC and burden accrual towards burdensome clusters.

Work Package 4 ‘Lifecourse prevention’:

4.1 Identify and characterise clusters of early life exposures (risk factors for early-onset, burdensome MLTC-M and sentinel conditions) and characterise population groups at risk of future MLTC-M in early-life (prebirth-18 years).
4.2 Identify critical time points and key lifecourse targets for MLTC-M prevention and model counterfactual prevention scenarios acting on combined risk factors at the specified timepoints (prebirth-18 years).

Cross WP3 and WP4 objectives:

4.3 Investigate the influence of sentinel conditions and sequence of accrual of wider determinants, conditions and burdensome factors in the development of early-onset, burdensome MLTC-M clusters.
4.4 Compare AI and causal inference modelling for potential early-life (0-18) ‘preventable moments’ trajectory, defined as time periods in the life course where there is an opportunity for intervention on risk factors and early determinants to prevent the development of MLTC-M, and exploring alternative trajectories based on models of policies/strategies/interventions and outcomes.

Work Package 5 ‘People, Policy, and Impact’:

5.1 Identify key stakeholders and engage them in: 1) exploring timepoints and targets to prevent/delay specified sentinel conditions and early-onset, burdensome MLTC-M, 2) opportunities to narrow health inequalities and, 3) optimal dissemination strategies and pathways to impact.
5.2 In partnership with the patient and public advisory board, engage with stakeholders to co-produce public health implementation recommendations based on our research, and produce a policy and practice engagement strategy to disseminate findings through multiple channels.

## Dataset summary

The majority of work within this Research Collaboration involves the use of two large, pseudonymised linked routine healthcare datasets and three anonymised linked birth cohort datasets. Work package 1 also involves the recruitment of individuals to take part in a Delphi study; the details of this are given in the relevant work package sections below.

### Longitudinal birth cohort studies datasets

The *Aberdeen Children of the 1950s (ACONF)* includes children born in Aberdeen, Scotland between 1950 and 1956, in total there are 12,150 cohort members, and participants were traced in their forties (2002) and linked to hospital and mental health admissions, maternity records, cancer registers, and death records. The *National Child Development Study (NCDS)* has followed all children born in England, Scotland and Wales in one week in 1958. In total 17,415 cohort members have been followed up over 12 sweeps – birth, 7, 11, 16, 23, 33, 42, 44, 46 50 and 55. The *1970 British Cohort Study (BCS70)* has followed all children born in England, Scotland, Wales and Northern Ireland in one week in 1970. In total 17,196 cohort members have been followed up over 10 sweeps – birth, 5, 10, 16, 26, 30, 34, 38, 42, 46. Both the NCDS and BCS70 have collected information on socioeconomics, family background, cognitive development, educational, employment, partnerships, fertility, health and health-related behaviour and can be linked to hospital episode statistics.

### Routine health datasets

The *Secure Anonymised Information Linkage (SAIL) Databank* contains a range of anonymised linked individual-level, population-scale data sources for the population of Wales, including approximately 3.1 million people. Linked SAIL data sources contain health, administrative and anonymised demographic/geographic information for the population of Wales, collected between 2000 and 2022. The *Clinical Practice Research Database (CPRD)* includes both CPRD GOLD and CPRD Aurum providing data on over 60 million people between 2010 and 2022 and over 45 million people available for data linkage in England. Data is collected from GP practices and primary care data fully coded electronic health records, and includes pharmacy and pathology records (Vision® or EMIS®).

## Methods, design and analysis for specific work packages

### Work package 1: ‘Burden and complexity’

The aim of this work package is to develop a deeper understanding of what ‘burdensomeness’ and ‘complexity’ mean to people living with early-onset MLTC-M, their carers and healthcare professionals, and to use this to inform data curation (WP2), AI analyses (WP3) and future preventative strategies (WP4/5). We will develop a list of burdensomeness/complexity attributes that might be identified and characterised in routine healthcare data as burdensomeness/complexity indicators. We will refine these indicators with people who live with (or care for people with) MLTC-M, healthcare professionals and MLTC-M experts and identify burden that might be prevented/reduced.

#### Qualitive evidence synthesis

We will conduct a qualitative evidence synthesis (QES) to collate findings of relevant qualitative studies on the experience of living with MLTC-M.^31^ We will summarise the scope/QES question using the ‘PerSPecTIF’ framework ^32^ and register the protocol on ‘PROSPERO’.^33^ Literature searching will follow Cochrane’s Handbook for Systematic Reviews of Interventions, ‘Qualitative Evidence’ guidance,^34^ and we will search Database including: MEDLINE (EBSCO), PsycINFO (EBSCO), CINAHL (EBSCO), Cochrane Library, EMBASE (EBSCOhost) as well as grey literature and backward/forward manual searching. Date restriction (post 2000) will be applied to ensure relevance, and inclusion criteria will include qualitative studies, studies exploring lived experience/management of multimorbidity and mixed methods studies with a qualitative element. Exclusion criteria include single-condition studies with comorbidities and studies in children (older adults are not excluded but age distribution of participants will be considered and reported). However, we anticipate the inclusion and exclusion criteria will be developed iteratively over the course of the study. Two researchers will screen independently, and a third will adjudicate conflicts.

Data extraction will be completed in two stages with the support of public contributors: Stage 1: contextual details (including population, context, methodology, recruitment, data collection, analysis), and Stage 2: individual study findings. Data synthesis methods will be finalised when the included studies are established.^35^ We will follow ‘RETREAT’ guidance to choose methodology.^36^ ‘Best Fit Framework Synthesis’ may be most appropriate as it adopts a predominantly deductive approach with a pre-specified framework while allowing inductive elements.^37,38^ The cumulative complexity model will be the framework as it is patient-centred.^22^ Thematic Synthesis or meta-ethnography will also be considered and we will learn from a review of qualitative systematic reviews.^39,40^ We will evaluate our QES using NVIVO (data management), CORE-Q (quality appraisal),^41^ ENTREQ (reporting)^42^ and GRADE CERQual (degree of confidence in our findings).^43^

#### Consensus study

Using findings from the QES, we will build consensus among multimorbidity experts and patients/carers of people with MLTC-M on:

• Which aspects of burdensomeness/complexity can be identified and characterised in routine healthcare data?
• Which aspects of burdensomeness/complexity might be reduced or prevented?
• Which indicators are most important to patients and carers?

The Delphi technique is widely used in LTC research.^44–46^ Ours will comprise three rounds. In round one, participants will independently rank a series of questions about potential indicators related to ‘burdensomeness’ and, ‘complexity’ using a four-point Likert scale. Health professionals/MLTC-M experts will indicate whether they believe the indicator can be characterised in routine data, and patients and carers will be asked to rank its importance to them. We will use a similar process to seek consensus on potentially preventable burdensomeness/complexity aspects from patient, carer and expert perspectives. Each domain will include free-text response sections, including opportunity to suggest other indicators that might be important to collect in the future. Panel recruitment will involve non-probability purposive sampling of about twelve healthcare professionals/MLTC-M experts and twelve adults who live with MLTC-M (or represent people who do) to achieve sufficient numbers and a range of participants by age, sex, ethnicity, profession, and geographical location.^47^ Participants will be primarily UK-based (as indicators will be applied in UK data). Participants will be required to respond across all rounds, but may withdraw at any time, and written, informed consent will be obtained from participants. Indicators and preventable aspects from the QES will be used to develop survey rounds.^46^ We will use study team, PPI Advisory Board and Expert Advisory group input to ensure clarity and ‘sense check’ questions, ensuring they cover aspects important to patients/carers. We will pilot the survey with iterative feedback to improve structure and readability. In Rounds 2 and 3, content will be iterated to incorporate qualitative comments from preceding stages, fed back in quantitative form. Consensus will be defined as >75% (the median level of agreement threshold from a systematic review of Delphi studies).^48^ of participants agreeing or disagreeing about:

1) Importance to patients,
2) Identifiability in routine data and,
3) Potential for prevention.

### Work package 2: ‘Trustworthy and responsible AI’

The aim of this work package is to design, build and operate a trustworthy and responsible environment for research into AI pipelines exploring early onset, burdensome MLTC-M using our routine and birth cohort datasets. By adopting and complying fully with principles defined by the DHSC AI Code of Conduct “A guide to good practice for digital and data-driven health technologies”^49^ and the “Five Safes” framework,^50^ We will ensure AI activities deliver benefits to society that are ethical, valuable, fair, safe, legal and conducted reflecting principles of open science including data/code transparency, interoperability, and standardisation. Equality, Diversity and Inclusion will be assessed relating to Public Sector Equality Duty under Section 149(1) and (3) of the Equality Act (2010).

#### Safe people, settings and data

We will address the need for “Safe People” by providing training to the research team in the guiding principles of the AI Code of Conduct and access to eLearning materials for clinical good practice, data security awareness and working with Trusted Research Environments (TREs). We will operate an information governance framework to manage data assets and associated legal and ethical risks, obtaining necessary approvals for storage, processing and linking. Having established “Safe data” through assessment of risk of reidentification and data owner constraints, curation pipelines will be provisioned in Swansea, Southampton, Aberdeen, UK Data Service (See Table 1).^51,52^ These nationally distributed TREs will provide “Safe Settings” to ensure data-related activities are undertaken securely and safely in accordance with data protection law and data licensing constraints. We will then explore processes supporting 1) cross-institutional collaboration for MELD-B researchers, 2) Machine Learning Operations (MLOps, experiment management, provenance tracking, containerisation, etc.) in each setting for reliable and repeatable data engineering and model development, and 3) federated learning, testing and validation of models.

**Table 1.** Trusted research environment and datasets.

TRE	Datasets
SAIL Databank, Swansea	SAIL linked data sources
NHS DSPT Compliant Secure Research Environment, Southampton	CPRD
Grampian Data Safe Haven, Aberdeen	ACONF record-linked to routinely available secondary care data
UK Data Service	BSC70 and NCDS record-linked to hospital episode statistics

#### Data readiness and curation

We will implement data engineering and curation processes to assess, prepare and harmonise data for analysis. MELD-B brings together routine and birth cohort data requiring development of reusable data engineering functions to prepare data such as metadata annotations, harmonisation of variables related to burdensomeness state space and generation of optimal data structures for analysis (e.g., timeseries events, graphs). We will develop specifications and tools to assess data readiness levels considering emerging data requirements from novel MELD- B algorithms for clustering, sequencing and counterfactual analyses. We will address challenges of accuracy, semantic consistency, missing data, bias and power, and implement processes for lifecourse burdensome state reconstruction by:

- Semantically aligning data with SNOMED CT medical terminology through existing mappings (e.g.,ICD10) and generate new mappings for prioritised variables in birth cohort data by processing metadata using semantic alignment and natural language processing.
- Profiling data to generate summary metadata including occurring patterns, frequencies and distributions, and distinct or missing values in a column, data types of attributes.
- Combining generated metadata with contextual information captured by WP1 structured in formal ontology to provide a contextualised metadata summary for reporting, audit and harmonisation across the AIM programme.

We will define a data quality model for analysis of lifecourse burdensomeness allowing systematic measurement and assessment against metrics for completeness, uniqueness, timeliness, validity, accuracy, and consistency. We will address bias and fairness as critical elements of model fairness and ensure outcomes do not discriminate against groups, and to clearly explain limitations in models. We will ensure that principles of fairness and responsible AI practices are adopted by everyone involved in data selection and algorithmic decisions. Bias will be addressed qualitatively by engaging stakeholders, guided by the expert advisory group. We will also address bias by applying a formal model of data pre-processing operations (feature selection, feature engineering, imputation and listwise deletion, resampling, outlier removal, smoothing/normalisation and encoding) to record operator’s effect on the data, logging operations during processing through code-instrumentation.

#### Safe outputs and open science

We will ensure FAIR (Findable, Accessible, Interoperable and Reusable) data stewardship, model curation and research integrity. AI pipelines will be developed using containers (Docker, Singularity) and described using container orchestration in accordance with MLOps best practice. Models will be developed in interactive environments such as Jupyter, tracked using an experiment manager (e.g., MLFlow) and built into software libraries for integration into workflows (e.g., TensorFlow). Artefacts such as software libraries, images, and notebooks will be made available through open-source licenses (subject to code review and safe output) allowing results to be replicated by others and research outputs repeated/compared by others. We expect to curate and make available metadata and data assets subject to governance and license constraints.

#### LTC inclusion

Clinical Co-Is will review code lists and agree LTC definition using existing clinical code lists including, but not limited to, 40 LTCs from a paper by Hanlon et al exploring associations between MLTC-M and adverse health outcomes in UK Biobank and the SAIL Databank, and 59 from a Delphi study by Ho et al that reached consensus on LTCs that should ‘always’ or ‘usually’ be included in MLTC-M analyses (noting that there is some overlap with LTCs in Hanlon et al).^53,54^ We will explore whether additional LTC codes from the CALIBER platform, Cambridge, and SAIL are needed as our definition of burdensome emerges.^55–57^ Clinicians will match birth cohort health variables to Read/SNOMED codes in routine data. We will then harmonise LTC definitions across BCS70/NCDS/ACONF/SAIL and CPRD where possible.

### Work package 3: ‘Sequencing and clustering’

The aim of this work package is to cluster individuals within the space of burdensomeness indicators and analyse determinants of clusters and sequence of acquisition of burdensome features for individuals in those clusters. Burdensome clusters will initially be identified in CPRD/SAIL that include information on later life. We will then develop methodology to connect birth cohort data with these clusters, permitting inference across the complementary birth cohort and routine datasets to identify early-life determinants of burden. Finally, we will identify and analyse the sequence of sentinel conditions and subsequent accrual of burden in harmonised birth cohort and routine data.

#### Clustering

We will apply AI technologies for clustering and cluster interpretation iteratively, with a human expert in the loop, on our routine GP datasets to elicit the structure of the burdensomeness space and the prevalent trajectories of evolution of MLTC-M within it. We will investigate how transience of health conditions and components of burden affect cluster stability, persistence and membership over time to inform our dynamical modelling of trajectories to burdensome MLTC-M. Methods to achieve this may include agglomerative, hierarchical, k-prototypes and graphical clustering, and XGBoost-SHAP cluster interpretation.

#### Inference across datasets

The birth cohort datasets contain much richer information on early life than the routine healthcare datasets; however birth cohort data are not linked to routine healthcare data and we do not know whether an individual represented in a birth cohort dataset appears in a routine healthcare dataset or vice versa. Therefore, to explore early-life predictors of burdensome MLTC-M we will identify individuals in our birth cohort datasets with patterns of clinical and social variables similar to those predicting burdensome cluster membership in the routine datasets. This will allow us to identify individuals in the birth cohort datasets who are likely to belong (now or in the future) to the burdensome clusters and use explainable AI methods and causal DAG-based models to determine early-life predictors of the burdensome clusters. We will use semi-supervised learning to classify individuals from our birth cohort datasets into the clusters identified in our routine datasets, starting from overlapping clinical and social variables recorded in the two types of datasets. The specific variables used to infer across datasets will be developed and refined throughout the project.

#### Lifecourse trajectories and dynamical modelling

We will explore the sequence of exposure to lifecourse risk factors, occurrence of the sentinel LTC and sequence of accrual of other LTCs and burdensome features in birth cohort and routine datasets, and characterise those that lead to early MLTC-M in the burdensome clusters. This may indicate how the order of acquisition of conditions influences the development of MLTC-M and suggest points of intervention. We will build dynamical models of the acquisition of LTCs, elements of burden and risk factors leading to burdensome MLTC-M and compare them with data on the dates of acquisition of these features by individuals in the routine and birth cohort datasets. We will investigate the risk of developing a given condition or burdensome element, conditional on the sequence and timing of prior acquisition of LTCs and aspects of burden over the lifecourse. By exploring counterfactual scenarios, such as a change in sequencing or timing of conditions or risk factors, we aim to identify key timepoints at which to address specific risk factors in the prevention of onset of the specified burdensome MLTC-M clusters. Methods to achieve this may include statistical multistate modelling and approximate Bayesian computation in combination with individual-based modelling.

### Work package 4: ‘Lifecourse prevention’

The aim of this work package is to identify the important early-life (from preconception, pregnancy, and birth to childhood and adolescent) characteristics of population groups at risk of future early-onset multimorbidity and use this characterisation to model the nature and timing of targeted public health prevention scenarios of early-onset, burdensome/complex MLTC-M through examining counterfactual scenarios of lower risk. We will do this through:

#### Characterisation of early health, social, economic and environmental risk factors

We will explore the early health, behavioural, social, economic and environmental characteristics of population groups and their risk of sentinel conditions and early-onset, burdensome MLTC-M. We will do this through describing clusters of early life exposures (potential determinants of early-onset, burdensome MLTC-M and sentinel conditions) and characterising their components, pattern, and time trend. We will start with a long list of candidate determinants from the three birth cohorts and SAIL Databank , and develop criteria for selection guided by a conceptual model, degree of association with target outcomes, public and patient input, scoping the relevant literature and policy directions.

#### Causal inference

Utilising a causal inference approach to visualise apriori knowledge and assumptions about what confounds and mediates relationships of interest we will explore adopting a directed acyclic graph (DAG)-based approach informed by the generated early life clusters based on the project data, prior evidence, biological plausibility and patient and public involvement. The outcomes will be informed by findings from work packages 1 and 3.

#### Identification of critical time-points for public health interventions

We will examine the nature and critical time-points for potential public health interventions of early-onset multimorbidity and the elements within clusters of risk factors that are most important to act on for early prevention. Using counterfactual scenarios of prevention, we will conduct comparative analysis to specify what is most effective in reducing early-onset multimorbidity risk; action on individual risk factors (classical public health approach) or simultaneous action on a combination of risk factors at specific time-points between pre-birth and 18 years. We will the explore the use of methods such as adjusted population attributable fractions or G-methods or which deal with time varying exposures to estimate the effect of hypothetical interventions at critical points before adulthood (pre-birth to 18 years) on the prevention of multimorbidity and burdensomeness.^58^ Such methods estimate potential outcomes under less restrictive conditions than standard regression methods and there is less risk of biased estimates due to over-adjustment.^59^ These also follow a DAG approach to represent causal assumptions and compare time-varying joint and dynamic interventions.

### Work Package 5: ‘People, policy, and impact’

The aim of this work package is to connect emerging findings with relevant stakeholders in order to identify appropriate intervention opportunities, effective means of dissemination and effective policy outputs. Through learning from each work package, and engagement and co-production with key stakeholders, we aim to identify prevention opportunities across the life course and establish pathways to policy and practice impact. We will establish a Policy and Practice Engagement Strategy and undertake:

1. Stakeholder analysis to identify key MLTC-M stakeholders and identify key stakeholders relevant to emerging findings.
2. Policy workshops to review burdensomeness/complexity and prioritising policy/practice outputs.
3. On-going stakeholder engagement, in partnership with the PPI Advisory Board.

These activities we will help us to prioritise populations, timepoints and determinants to target for the prevention/delay of specified sentinel conditions and early-onset, burdensome MLTC-M, and to identify opportunities to narrow health inequalities. We will also determine the most effective ways to disseminate findings and ensure pathways to impact. Finally, we will co-produce public health and practice implementation recommendations based on this research and determined by its findings.

#### PPI advisory board

Our PPI lead will, along with a PPI Officer, will lead the PPIE structure in MELD-B, with a team of PPIE contributors forming a Patient and Public Advisory Board. We will seek diversity among PPIE contributors, considering age, sex, multimorbidity experience, ethnicity, and background, and contributors will be drawn from across our collaboration sites. The PPI Advisory Board will meet regularly, and meetings will be chaired by our PPI lead, and co-chaired by a PPI member. PPI members will be paid according to the NIHR payment guidance.^60^ We will create a safe, supportive environment for PPI contributors to bring their knowledge and experience. In addition, we will work with PPI groups in Glasgow, which is an ideal context to explore multimorbidity and socioeconomic deprivation. We will also consult with the public and communities beyond the PPI Board and Glasgow groups as guided by our developing findings and objectives, linking into diverse communities and networks.

## Outputs

**Figure 2.**
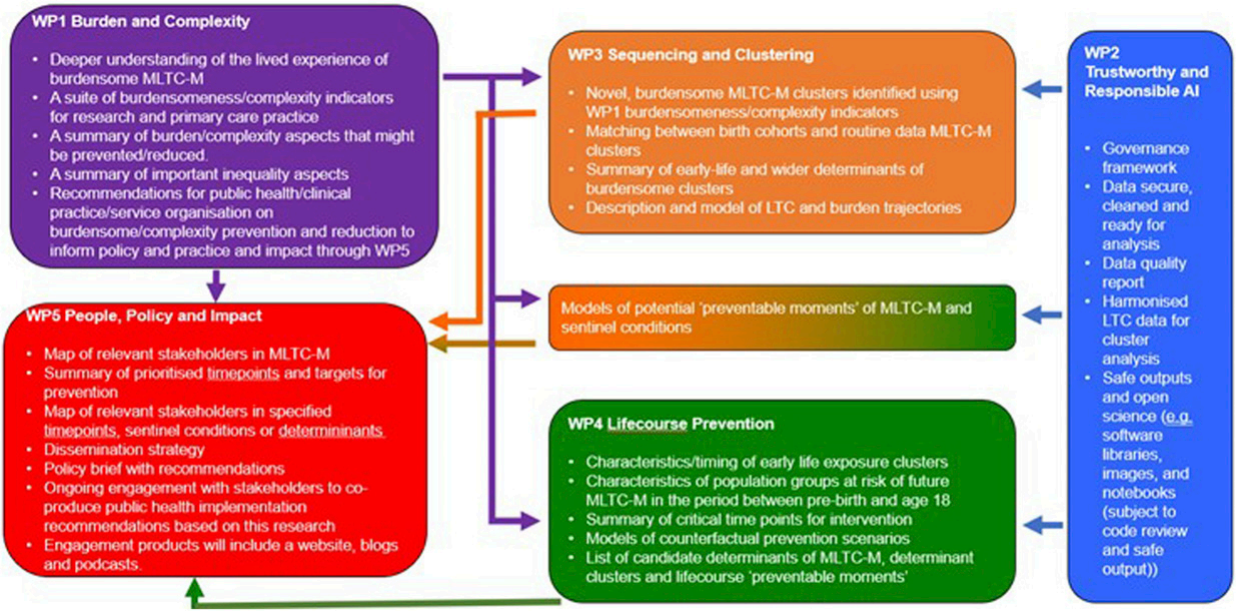
MELD-B research collaboration outputs by work package.

The outputs we aim to generate from the MELD-B Research Collaboration are summarised in Figure 2.

## Ethics approvals and dissemination

The study will be conducted in accordance with the UK Policy Framework for Health and Social Care Research. Ethics approval has been obtained from the University of Southampton Faculty of Medicine Ethics committee (ERGO II Reference 66810).

The regulatory authorities will be notified of any subsequent non-substantial amendments deemed necessary for the study. The appropriate approvals will be sought for any substantial amendments, and the appropriate approvals and processes will be followed for linked datasets (NCDS/BCS70/ACONF).

The dissemination of our findings will be achieved through academic (conferences, academic papers, harmonising datasets for wider user and working with other award holders) and non-academic (social media, website, blog and a podcast) channels. Central to our dissemination plans is to ensure that our findings have maximum impact, particularly to non-academic audiences. We will undertake broad engagement activities with our Patient and Public Advisory Board and other key stakeholders to identify potential for impact from outputs. Our extensive connections in local and national Public Health will help facilitate this.

## Discussion/conclusion

### Challenges

The MELD-B research collaboration will face several practical and operational challenges, we recognise that new challenges will emerge and change as the project progresses, and it is not possible to anticipate every eventuality. However, challenges include gaining access to five separate datasets, harmonisation and volume- and quality- control of variables across these datasets. Methodological challenges include ensuring we utilise the most appropriate methods to use as these are likely to vary depending on the questions being addressed through the life of the project as new ideas emerge. It is also important we ensure that our Patient and Public Advisory Board are embedded in the project from its conception and that PPI colleagues are supported to provide meaningful contributions across all work packages. The development and understanding of new definitions and terminology across disciplines of public health, epidemiology, data science and computer science and engaging relevant stakeholders before we have findings will also be challenging. Additionally, an important aspect we face is the scale of the project – we are likely to have a team of over 40 members, across multiple locations and institutions in the UK, with different skills sets and disciplines who are working together to achieve multiple objectives in a short space of time. Therefore, managing the dynamics associated with such a large, complex project is a recognised issue.

### Potential impact

Traditional models of prevention (primary, secondary, tertiary) are limited in application to multimorbidity, particularly when considering the accrual of conditions over the lifecourse. The Academy of Medical Sciences emphasised prevention of early-onset MLTC-M clusters and targeting individual risk and its research priorities included identifying ‘the burden caused by common clusters of conditions’.^61^ It highlighted conditions in common clusters with much less evidence on prevention, such as depression.^61,62^ Public Health England has highlighted the importance of a lifecourse approach and identifying critical points for intervention: ‘Unlike a disease-oriented approach, which focuses on interventions for a single condition often at a single life stage, a lifecourse approach considers the critical stages, transitions, and settings where large differences can be made in promoting or restoring health and wellbeing.’^63^ Moreover, public health interventions focusing on wider determinants are highly cost-effective, averaging a fivefold return on investment.^64^ From a public health perspective, characterising the sequence of accrual of conditions and their determinants across the whole lifecourse would give the opportunity to identify critical timepoints for population-level prevention efforts.

In summary, the MELD-B Research Collaboration aims to fill several key gaps in the research evidence in MLTC-M and thereby influence policy and practice. It will achieve this by developing a deeper understanding of the lived experience of ‘burdensomeness’ and ‘complexity’ of multimorbidity, identifying new clusters of burdensome MLTC-M and their key early-life risk factors, mapping trajectories across the lifecourse towards burdensome clusters in those under 65, and modelling prevention scenarios to inform policy.
